# Notes and Notices

**Published:** 1896-09

**Authors:** 


### NOTES AND NOTICES.

Dr. L. D. Rogers and Dr. Ida Wright Rogers, of Chicago, have returned from a visit to the principal hospitals of England, Ireland, Scotland, France, Switzerland, Southern Germany, Northern Germany, and Belgium.

The former was graduated last year from Rush Medical College, and was the first alumnus of that Institution to have his literary and medical degrees recognized by the Royal College of Physicians of London, and the Royal College of Surgeons of England.

A Rare Effect of Tobacco.—J. W. Scott, M. D., reports a case of convulsions, epileptiform in character, due to the use of tobacco. For two months he had one or two convulsions a week, and they were growing progressively worse in spite of treatment. With the discontinuance of tobacco the convulsions ceased, and have not returned.—*Southwestern Medical Record.*

No Marriage Without Vaccination.—The stringency of the vaccination laws is in England the subject of noisy agitation on the part of ignorant demogogues; but what would some persons say to the interference with the rights of the individual which is maintained by the laws of Norway and Sweden? In these countries, so impressed is the legislature not only with the advantages, but with the public duty of vaccination that before a couple is legally married certificates must be produced showing that both the bride and bridegroom have been satisfactorily vaccinated.—*British Medical Journal in the Texas Medical News.*

The Proceedings of the International Hahnemannian Association, session of 1896, are in print, and will be delivered on October 1st to all members whose dues are paid for the current year. Erastus E. Case, Secretary.

